# Minimal disease activity (MDA) in patients with recent-onset psoriatic arthritis: predictive model based on machine learning

**DOI:** 10.1186/s13075-022-02838-2

**Published:** 2022-06-24

**Authors:** Rubén Queiro, Daniel Seoane-Mato, Ana Laiz, Eva Galíndez Agirregoikoa, Carlos Montilla, Hye-Sang Park, Jose A. Pinto-Tasende, Juan J. Bethencourt Baute, Beatriz Joven Ibáñez, Elide Toniolo, Julio Ramírez, Ana Serrano García, on behalf of Proyecto REAPSER Study Group, Juan D. Cañete, Xavier Juanola, Jordi Fiter, Jordi Gratacós, Jesús Rodriguez-Moreno, Jaime Notario Rosa, Andrés Lorenzo Martín, Anahy Brandy García, Pablo Coto Segura, Anna López Ferrer, Silvia Pérez Barrio, Andrés J. Plata Izquierdo, Sagrario Bustabad, Francisco J. Guimerá Martín-Neda, Eduardo Fonseca Capdevilla, Raquel Rivera Díaz, Andrea Cuervo, Mercè Alsina Gibert, Pilar Trenor Larraz, Isabel de la Morena Barrio, Laura Puchades Lanza, Diego Bedoya Sanchís, Catalina Meliá Mesquida, Claudia Murillo, Manuel J. Moreno Ramos, María D. Beteta, Paloma Sánchez-Pedreño Guillén, Leticia Lojo Oliveira, Teresa Navío Marco, Laura Cebrián, Pablo de la Cueva Dobao, Martina Steiner, Santiago Muñoz-Fernández, Ricardo Valverde Garrido, Manuel León, Esteban Rubio, Alejandro Muñoz Jiménez, Lourdes Rodríguez Fernández-Freire, Julio Medina Luezas, María D. Sánchez-González, Carolina Sanz Muñoz, José M. Senabre, José C. Rosas, Gregorio Santos Soler, Francisco J. Mataix Díaz, Juan C. Nieto-González, Carlos González, Juan G. Ovalles Bonilla, Ofelia Baniandrés Rodríguez, Fco Javier Nóvoa Medina, Dunia Luján, María D. Ruiz Montesino, Ana M. Carrizosa Esquivel, Cristina Fernández-Carballido, María P. Martínez-Vidal, Laura García Fernández, Vega Jovani, Rocío Caño Alameda, Silvia Gómez Sabater, Isabel Belinchón Romero, Ana Urruticoechea-Arana, Marta Serra Torres, Raquel Almodóvar, José L. López Estebaranz, María D. López Montilla, Antonio Vélez García-Nieto

**Affiliations:** 1 Rheumatology Service & the Principality of Asturias Institute for Health Research (ISPA), Faculty of Medicine, Universidad de Oviedo, Oviedo, Spain; 2 Research Unit, Spanish Society of Rheumatology, Madrid, Spain; 3 Rheumatology and Autoimmune Disease Department, Hospital Universitari de la Santa Creu i Sant Pau, Barcelona, Spain; 4 Rheumatology Service, Hospital Universitario Basurto, Bilbao, Spain; 5 Rheumatology Service, Hospital Universitario de Salamanca, Salamanca, Spain; 6 Rheumatology Service-INIBIC, Complexo Hospitalario Universitario de A Coruña, A Coruña, Spain; 7 Rheumatology Service, Hospital Universitario de Canarias, Sta. Cruz de Tenerife, Spain; 8 Rheumatology Service, Hospital Universitario 12 de Octubre, Madrid, Spain; 9 Rheumatology Service, Hospital Universitari Son Llàtzer, Palma de Mallorca, Spain; 10 Arthritis Unit, Rheumatology Department, Hospital Clínic Barcelona, Barcelona, Spain; 11 Knowledge Engineering Institute, Universidad Autónoma de Madrid, Madrid, Spain

**Keywords:** Recent-onset psoriatic arthritis, Minimal disease activity, Predictive model, Machine learning

## Abstract

**Background:**

Very few data are available on predictors of minimal disease activity (MDA) in patients with recent-onset psoriatic arthritis (PsA). Such data are crucial, since the therapeutic measures used to change the adverse course of PsA are more likely to succeed if we intervene early. In the present study, we used predictive models based on machine learning to detect variables associated with achieving MDA in patients with recent-onset PsA.

**Methods:**

We performed a multicenter observational prospective study (2-year follow-up, regular annual visits). The study population comprised patients aged ≥18 years who fulfilled the CASPAR criteria and less than 2 years since the onset of symptoms. The dataset contained data for the independent variables from the baseline visit and from follow-up visit number 1. These were matched with the outcome measures from follow-up visits 1 and 2, respectively. We trained a random forest–type machine learning algorithm to analyze the association between the outcome measure and the variables selected in the bivariate analysis. In order to understand how the model uses the variables to make its predictions, we applied the SHAP technique. We used a confusion matrix to visualize the performance of the model.

**Results:**

The sample comprised 158 patients. 55.5% and 58.3% of the patients had MDA at the first and second follow-up visit, respectively. In our model, the variables with the greatest predictive ability were global pain, impact of the disease (PsAID), patient global assessment of disease, and physical function (HAQ-Disability Index). The percentage of hits in the confusion matrix was 85.94%.

**Conclusions:**

A key objective in the management of PsA should be control of pain, which is not always associated with inflammatory burden, and the establishment of measures to better control the various domains of PsA.

**Supplementary Information:**

The online version contains supplementary material available at 10.1186/s13075-022-02838-2.

## Background

Psoriatic arthritis (PsA) is a chronic inflammatory disease that can affect up to one-third of patients with psoriasis. In Spain, approximately 0.6% of the adult population has PsA (i.e., more than 200,000 affected patients) [1]. PsA is characterized by its potential for manifesting as multiple musculoskeletal and cutaneous-nail conditions that may overlap and negatively affect physical functioning and quality of life [2].

The treatment objectives recommended by the European League Against Rheumatism (EULAR) are remission assessed using the Disease Activity in Psoriatic Arthritis (DAPSA) score or minimum inflammatory disease evaluated using the minimal disease activity (MDA) score [3]. The latter, therefore, is not a continuous measure of activity but a dichotomous treatment objective (i.e., either it is reached or it is not). The MDA score comprises 7 relevant disease-related domains (tender/swollen joint count, skin, physical function, pain, enthesis, general patient-based evaluation), such that achieving 5 of the 7 indicates MDA, whereas achieving 7/7 indicates very low disease activity, which is similar to remission [4].

One aspect of particular interest in the MDA response is the evaluation of those variables that are associated with a greater or lesser probability of meeting this therapeutic objective. Registries and observational studies have revealed that younger age, male sex, better baseline physical function, lower baseline activity level, shorter disease duration, and better overall well-being are associated with a greater probability of achieving MDA after treatment with anti-TNF agents [4]. In addition, some comorbid conditions, especially those involved in metabolic syndrome, have been associated with a lower probability of reaching MDA, whereas weight loss strategies increase the possibility of meeting this objective after treatment with anti-TNF agents [4]. It is relevant that patients who achieve MDA, especially when this response is maintained over time, develop less structural damage and can better control the early atherosclerosis that accompanies the disease [4, 5].

At present, very few data are available on predictors of MDA in patients with recent-onset PsA. Such data are crucial, since the therapeutic measures used to change the adverse course of PsA are more likely to succeed if we intervene early. In the present study, we used predictive models based on artificial intelligence to detect variables associated with achieving MDA in patients with recent-onset PsA.

## Methods

The design of the REAPSER study has been described in detail elsewhere [6].

It is a multicenter observational prospective study (2-year follow-up, regular annual visits) promoted by the Spanish Society of Rheumatology. The study population comprised patients of both sexes aged ≥18 years who fulfilled the Classification Criteria for Psoriatic Arthritis (CASPAR) [7], with less than 2 years since the onset of symptoms attributable to the disease.

The intention at the baseline visit was to reflect the patient’s situation before disease progress was modified by the treatments prescribed in the rheumatology department. In this sense, participants could not have been receiving methotrexate, leflunomide, or apremilast for more than 3 weeks after initiation and could not be receiving biologic disease-modifying antirheumatic drugs (DMARDs). These intervals were fixed taking into account the fact that the mean time from initiation of treatment until onset of the response to therapy is 4 weeks in the case of synthetic DMARDs and 1 week in the case of biologic DMARDs. In cases where the patient had been receiving synthetic DMARDs for more than 3 weeks, we obtained confirmation from the investigating rheumatologist that the patient had not yet responded to treatment at the baseline visit; this information was sought in only 9 patients, and for all those involved, the time since initiation of synthetic DMARDs was under 2 months.

If patients with psoriasis receiving treatment with synthetic or biologic DMARDs developed PsA and were referred to the rheumatology department for diagnosis and management, then they could be included in the study, since this would not violate the criterion that the baseline visit reflected the situation of the patient before disease progress was modified by the treatment prescribed at the rheumatology clinic.

Since this was an observational study, in the follow-up visits participants were treated according to clinical practice.

Patients were consecutively invited to participate at one of their scheduled visits to the rheumatologist. Recruitment began in November 2014 and ended in October 2016. The second follow-up visit for the last patient was in December 2018. A total of 25 centers from 11 of the 17 Spanish autonomous communities participated in the study.

All patients gave their informed consent to participate. The study centers assigned each patient an identification code in order to ensure data confidentiality in line with current legislation. The study was approved by the Clinical Research Ethics Committees of the Principality of Asturias (study number 14/2014).

### Variables and measurement

- *Sociodemographic data*: age; sex (1. male, 2. female); educational level (none, primary, secondary, university).
- *Family history (father, mother, grandparents, siblings, children)* of PsA, other types of inflammatory arthritis, and psoriasis.
- *Personal history and comorbidities (based on a review of medical records)*: age-adjusted Charlson comorbidity index, cardiovascular risk factors (arterial hypertension, hyperlipidemia, diabetes mellitus [differentiating between insulin and non-insulin-dependent]) [8].
- *Anthropometric data*: Body mass index (BMI).
- *Lifestyle*: smoking (patients who reported having smoked at least 100 cigarettes in their lifetime and who at the time of the visit smoked every day or on some days were classified as “current smoker.” Patients who reported having smoked at least 100 cigarettes throughout their lifetime and who at the time of the visit did not smoke at all were classified as “ex-smokers”. Patients who reported not having smoked 100 cigarettes were defined as “never smokers”). Alcohol consumption was measured in standard alcohol units per week and evaluated using the Systematic Interview of Alcohol Consumption) [9]. Physical activity was evaluated using the short form of the International Physical Activity Questionnaire (IPAQ); 3 levels were established for the analysis (low, moderate, and high), according to the guidelines for data processing and analysis of the IPAQ [10].
- *Clinical situation at diagnosis of PsA*: year of presentation of PsA symptoms; clinical form (1. axial, 2. peripheral. 3. mixed); articular pattern (1. oligoarticular, 2. polyarticular, 3. distal, 4. mutilans, 5. spondylitis); presence of dactylitis (yes/no).
- *Joint involvement and enthesitis*: number of tender joints (NTJ68); number of swollen joints (NSJ66); extended version of the Maastricht Ankylosing Spondylitis Enthesitis Score (MASES) [11]. Polyarthritis was defined as NSJ66 ≥5.
- *Pain and global assessment of disease during the previous week*: Patient global pain on a scale ranging from 0 (no pain) to 10 (very intense); patient global assessment of disease on a scale ranging from 0 (feels very well) to 10 (feels very ill); physician global assessment of the disease on a scale ranging from 0 (minimal activity) to 10 (maximum activity).
- *Cutaneous and nail involvement (evaluated by a dermatologist)*: cutaneous psoriasis (yes/no); year of onset of psoriasis; clinical type (psoriasis vulgaris [plaques], guttate, erythrodermic, generalized pustular, localized pustular, inverse, other); specific locations (scalp, nails, palms and soles, gluteal cleft and/or perianal region, palmoplantar pustulosis, mucosal involvement); treatment of psoriasis and year of onset (topical treatment, phototherapy, retinoids, methotrexate, cyclosporine, etanercept, infliximab, adalimumab, ustekinumab, other). Body surface area (BSA) affected by psoriasis or Psoriasis Area and Severity Index (PASI) [12]; onychopathy (number of digits affected). For purposes of the analysis, severe psoriasis was defined as PASI >10.
- *Functional situation and quality of life*: Health Assessment Questionnaire (HAQ) [13], Psoriatic Arthritis Impact of Disease (PsAID) [14].
- *Radiologic evaluation at baseline*: Bath Ankylosing Spondylitis Radiology Index (BASRI) of the sacroiliac region [15], hand involvement according to the modified Steinbrocker method for PsA [16].
- *Laboratory tests*: C-reactive protein (CRP), uric acid, total cholesterol, LDL cholesterol, triglycerides. For purposes of the analysis, a series of cut-off points were established to define high values: >0.5 mg/dl for standard CRP; >0.3 mg/dl for high-sensitivity CRP; hyperuricemia if >7 mg/dl in men and >6 mg/dl in women; ≥200 mg/dl for total cholesterol; ≥100 mg/dl for LDL; ≥150 mg/dl for triglycerides.
- *Treatment of PsA with DMARDs, date of initiation, date of finalization*: synthetic DMARDs (methotrexate, leflunomide, sulfasalazine, apremilast, cyclosporine) and biologic DMARDs (adalimumab, etanercept, infliximab, golimumab, ustekinumab, certolizumab, secukinumab).

Minimum activity was defined as fulfillment of at least 5 of the following 7 criteria: ≤1 tender joint; ≤1 swollen joint; PASI ≤1 or BSA ≤3%; score on the visual analog scale (VAS) for pain provided by the patient ≤1.5; overall score for disease activity provided by the patient ≤2; HAQ score ≤0.5; ≤1 painful enthesis [17].

### Sample size

REAPSER study was planned as a registry intended to collect a large number of variables, without prespecified hypothesis. The initial estimation of recruitment in the REAPSER cohort was 295 patients, assuming that up to 25% could be lost to follow-up. This sample size would make it possible to detect as significant a relative risk >2.30, assuming an exposure of 50% (conservative assumption to maximize the required sample size), confidence level of 95%, and statistical power of 80%.

### Statistical analysis

#### Imputation of missing data

- The duration of psoriasis was imputed with the median of the remaining patients from the same age range. The age ranges used were as follows: <41 years, 41-60 years, and >60 years.
- Systemic treatment of psoriasis was imputed with 0 (that is, not receiving systemic treatment).
- Radiological involvement of the hands at the baseline visit was not imputed, except for those patients with an NPJ28 and NSJ28 value of 0, in which case it was imputed with 0.
- For patients who stopped attending the visits owing to improvement of their condition, the missing values for the variables PsAID and HAQ were imputed with 0. Minimum activity was imputed as present.

#### Generation of the dataset

The analysis was performed to determine predictive ability, attempting to establish associations between the outcome measures and values at the previous visit for the remaining variables. To do so, the dataset contained data for the independent variables from the baseline visit and from follow-up visit number 1. These were matched with the outcome measures from follow-up visits 1 and 2, respectively. Atemporal variables such as sex and family history were matched with outcome measures from follow-up visits 1 and 2; therefore, their values are the same for each one. This was also true for variables that were only collected at the baseline visit, such as systemic treatment of psoriasis at PsA diagnosis and PsA clinical form at diagnosis.

#### Bivariate analysis

We selected variables whose Spearman correlation was considered significant according to the threshold applied to the ρ correlation coefficient ($\mid p\mid >\frac{2}{\surd N}$, with N being the number of data items). We also applied methods based on artificial intelligence, specifically the XGBoost algorithm and the SHAP technique, in order to identify informative variables (See Additional file 1 for a detailed explanation of both approaches). Finally, of the variables identified in the previous steps, we selected those that were statistically significantly associated with the outcome measure (*p*<0.05). To do so, we applied the Mann-Whitney test for continuous/discrete variables and the *χ*^2^ test for categorical variables.

#### Multivariate analysis

We then trained a random forest–type machine learning algorithm to analyze the association between the outcome measure and the variables selected in the bivariate analysis (see Additional file 1 for more detail). The machine learning models are trained with 75% of the sample. When the samples generated are imbalanced, the models are trained using the oversampling technique, which is based on duplicating or triplicating data whose value for the outcome measure is a minority value.

In order to understand how the model uses the variables to make its predictions, we applied the SHAP technique (see Additional file 1 for more detail). This approach assigns a SHAP value to each value of each variable according to the extent to which it affects the prediction of the model (the higher the absolute SHAP value, the greater the influence of this data item on prediction) and to how it affects the prediction (if the SHAP value is positive, the data item positively affects the prediction, that is, it confers a higher value on the prediction). The SHAP summary graphs order the predictors by their importance in the predictions of the model. This importance is calculated with the mean of the SHAP values assigned to each data item of a variable; mean values <0.01 were considered to indicate the low importance of the variable in the model.

Furthermore, the functioning of the model is evaluated using the 25% of the data that were not used during training. The division between 75% for training and 25% for evaluation is generated in such a way that the proportion of each class of the outcome measure is the same in both datasets.

The performance of the model was visualized using a confusion matrix in the evaluation sample. This matrix shows the real class of the data items, together with the predicted class, and records the number of hits and misses.

## Results

The sample eventually comprised 158 patients. Table 1 summarizes the baseline characteristics.

**Table 1:** Baseline characteristics of the sample

Variable	
Age	49.35 (13.53)
Sex	
Male	90 (57%)
Female	68 (43%)
Educational level	
None	3 (1.9%)
Primary	58 (36.7%)
Secondary	66 (41.8%)
University	31 (19.6%)
BMI	27.63 (5.27)
Smoking	
Never smoked	61 (38.6%)
Ex-smoker	44 (27.8%)
Occasional smoker	6 (3.8%)
Daily smoker	47 (29.7%)
Weekly alcohol consumption	0 (0;4)
Family history of psoriasis	62 (39.2%)
Family history of psoriatic arthritis and other types of inflammatory arthritis	21 (13.3%)
Age-adjusted Charlson comorbidity index	1 (0;2)
Arterial hypertension	39 (24.7%)
Hyperlipidemia	53 (33.5%)
Diabetes mellitus	
Non-insulin-dependent	13 (8.2%)
Insulin-dependent	3 (1.9%)
Psoriasis	149 (94.3%)
Duration of psoriasis until onset of PsA (years)	10 (2;20)
Clinical form of psoriasis	
Vulgaris	126 (80.3%)
Guttate	5 (3.2%)
Localized pustular	10 (6.4%)
Inverse	7 (4.5%)
Psoriasis specific sites	
Scalp	88 (59.5%)
Nails	91 (61.5%)
Palms and soles	13 (8.8%)
Gluteal cleft and/or perianal region	34 (23.0%)
Mucous membranes	1 (0.7%)
PASI	1.2 (0.3;3.1)
Systemic treatment of psoriasis	21 (14.3%)
Clinical form of PsA	
Axial	12 (7.6%)
Peripheral	126 (79.7%)
Mixed	20 (12.7%)
Joint pattern in PsA	
Oligoarticular	87 (55.1%)
Polyarticular	47 (29.7%)
Distal	9 (5.7%)
Spondylitis	15 (9.5%)
Dactylitis at diagnosis	71 (44.9%)
Enthesitis at diagnosis	43 (27.2%)
Uveitis at diagnosis	1 (0.6%)
Pain in the previous week	5 (3;7)
Patient global assessment of disease	5 (3;7)
PsAID	3.75 (1.65;5.90)
Sacroiliac involvement (BASRI)	0 (0;1)
Hand involvement (modified Steinbrocker)	0 (0;2)

Thirty-three patients (20.9%) were lost to follow-up. For 10 of them, the investigating rheumatologist at their centers confirmed that they had not attended the visit because their PsA had improved.

At the first follow-up visit, 55.5% of the patients who attended the clinic had MDA. This percentage was 58.3% at the second follow-up visit.

Categorical variables are expressed as *n* (%). Numerical variables are expressed as mean (SD) if approximately normally distributed and as median (IQR) if not.

### Bivariate analysis

Table 2 shows the variables selected in the bivariate analysis.

**Table 2:** Variables associated with minimal disease activity of PsA: bivariate analysis

Variable	***P*** value
Sex	0.015
Weekly alcohol consumption	0.03
Joint pattern at diagnosis	0.01
Number of tender joints	0.01
Global pain	<0.001
Physician global assessment of disease	<0.001
Patient global assessment of disease	<0.001
PsAID score	<0.001
HAQ score	<0.001

### Multivariate analysis

The number of observations for the multivariate analysis was 256.

**Fig. 1:**
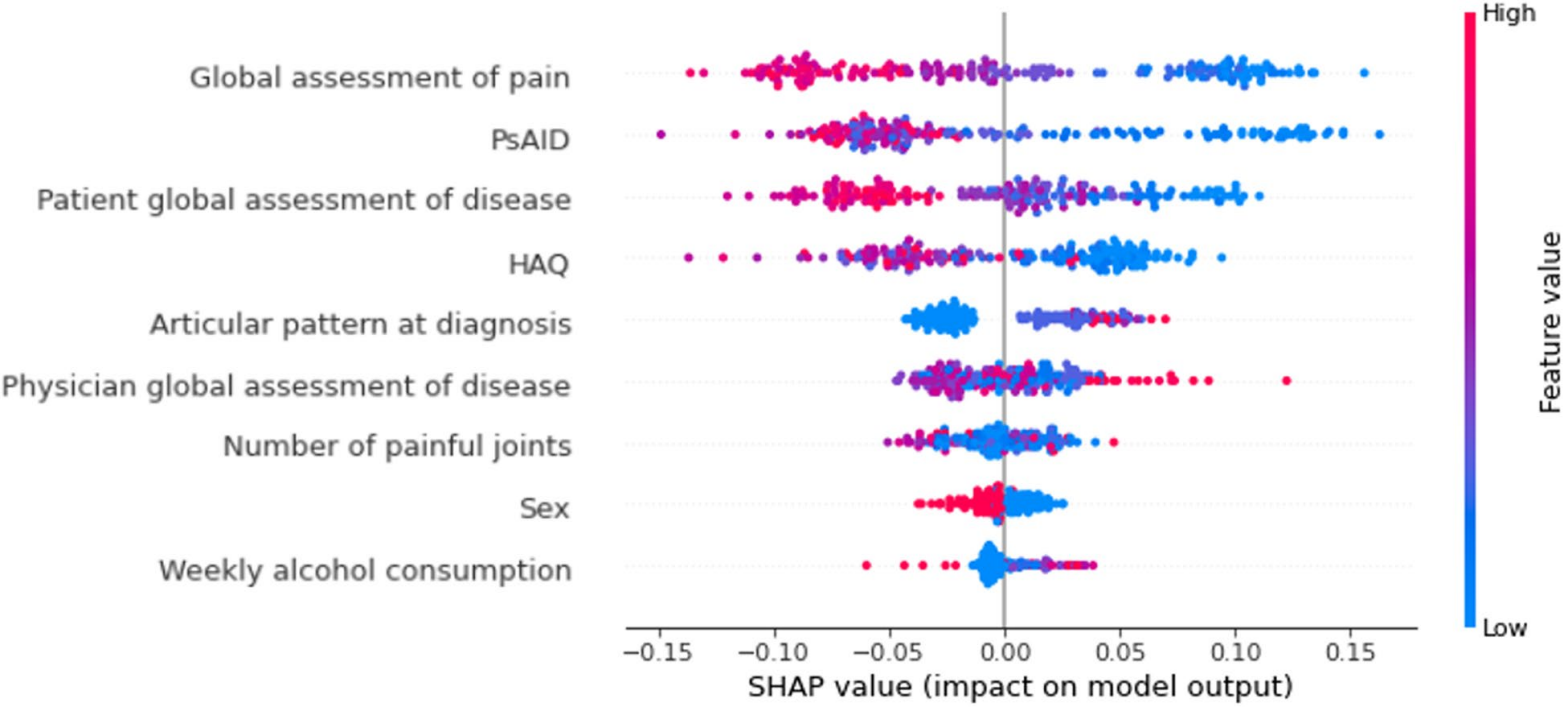
Random forest–type machine learning algorithm. SHAP summary graph

The SHAP values for each value of each variable are shown in Fig. 1. The vast majority of low values (blue points) in the global pain, PsAID score, patient global assessment of disease, and HAQ score are found in the positive part of the SHAP values, thus indicating that the direction of association between these variables and minimum activity was negative. Being female (highest value on the coding for sex, represented in red in Fig. 1) and the oligoarticular pattern (lowest value in the coding for articular pattern at diagnosis) were associated with a lower probability of minimal activity. For the remaining variables, the direction of the association was less clear.

The importance of the variables in the model according to the mean of the SHAP values is shown in Table 3. The variables that made the largest contribution to the predictions in the random forest were global pain and PsAID; those that made the smallest contribution were sex and weekly consumption of alcohol.

**Table 3:** Variables in the predictions of the random forest for MDA according to the SHAP method

Variable	Importance according to SHAP^**a**^
Global pain	0.069
PsAID	0.064
Patient global assessment of disease	0.047
HAQ	0.044
Articular pattern at diagnosis	0.029
Physician global assessment of disease	0.023
Tender joint count	0.014
Sex	0.009
Weekly alcohol consumption	0.009

*MDA* minimal disease activity

^a^Mean of the SHAP values for each value of the variable

Table 4 shows the confusion matrix representing the functioning of the random forest trained for prediction of minimal activity in PsA according to the 9 variables. The percentage of hits in the assessment sample was 85.94% (55/64).

**Table 4:** Functioning of the random forest model trained to predict minimal activity. Confusion matrix

	Minimal activity (predicted)	
	No	Yes
**Minimal activity (real)**		
**No**	23	7
**Yes**	2	32

## Discussion

In this multicenter prospective study carried out in patients with recent-onset PsA, assessed at baseline before the potential modification of its natural history because of the treatment prescribed by a rheumatologist, 9 disease variables were associated with achieving MDA. Of these, global pain, PsAID, patient global assessment of disease, and HAQ carried considerable predictive weight. Oligoarticular forms at onset and female sex were associated with a lower probability of achieving MDA. The direction of the association was less clear for the remaining variables. The confusion matrix representing the functioning of the random forest trained to predict MDA according to the 9 variables revealed a high hit rate (close to 86%), indicating the good performance of the model.

The first 2 variables in order of importance revealed by our artificial intelligence model were patient global pain and PsAID score. Pain is the disease domain to which patients usually attach greater weight when scoring both the activity and the impact of the disease [18]. In fact, the variable related to pain in the PsAID questionnaire is corrected by multiplying the VAS pain result by a factor of 3 [14].

Given that the MDA score contains 2 pain-specific items (tender joint count and global pain) and that the main variables in a PsAID score indicating high impact of disease are pain and high HAQ score, it is not surprising that the 2 main predictive variables for MDA are pain and PsAID score [4, 17, 19]. The contribution of our study lies in its prospective design, in such a way that independent variables were measured 1 year before MDA was assessed. Furthermore, the value of this analysis, beyond ascertaining which variables predict MDA, lies in our assessment of the order of importance of this prediction. In this sense, it is worth mentioning that in our model, global pain was considerably more important than tender joint count.

The next variables in order of importance in the predictive model were, once again, 2 variables that form part of the MDA response itself, that is, patient global assessment of disease and HAQ score. As expected, the direction of the association was once again negative. More recent literature reviews on the association between these 2 variables and MDA revealed a clear link in different scenarios (randomized clinical trials, registries, observational studies), so that the poorer the assessment by the patient and the poorer the physical function by HAQ, the greater the probability that the patients do not achieve MDA [4, 19, 20]. In fact, the kappa agreement between HAQ and MDA is usually better than the agreement between the latter and PsAID or remission according to DAPSA, which seems to confer a special value on worse baseline physical function for the probability of not achieving MDA [21]. Therefore, it is more difficult for patients with established and more advanced disease (especially if structural damage is already present) to achieve MDA, even if they do not currently have a high inflammatory burden [4, 19]. In any case, the observation mentioned above has been reported in cross-sectional observational studies, whereas in the present prospective study of patients with recent-onset PsA, PsAID score and patient global pain carry more predictive weight than the HAQ.

Despite its low predictive value in the model, female sex was associated with a reduced probability of achieving MDA. In general, almost all studies on the association between spondyloarthritis (including PsA) and sex find greater diagnostic delays in women (a factor clearly linked to poorer subsequent disease course and worse rates of response to therapy), higher levels of pain, poorer response to and persistence of biologics, and greater psychological distress, all of which necessarily entail poorer quality of life [4, 22]. These aspects somehow come together, making female sex a classic factor that is associated with poorer outcomes in PsA, including a lower probability of achieving MDA both in patients with early disease and established disease [4].

In contrast with data published in other settings, we found a negative association with MDA in patients with an oligoarticular pattern at diagnosis. A priori, this may seem counterintuitive, since outcomes are traditionally poorer in patients with a higher articular inflammatory burden (≥5 inflamed joints at baseline) [23]. Nevertheless, if we analyze the variables that make up the MDA score, we can see that major components of this response, such as VAS for global pain, HAQ, and patient global assessment, are not necessarily directly associated with inflamed joint counts [4, 19]. Moreover, a recent study comparing the components of the MDA response according to the patients’ PsAID, i.e., low or high impact, found that most patients in both situations fulfill the criterion of ≤1 inflamed joint [19]. That is, patients attribute a high impact to characteristics or consequences of the disease that have nothing to do with the inflammatory burden, as represented by the number of inflamed joints. In fact, it was not possible to establish a clear association between CRP and MDA [4]. Patients with a very low number of inflamed joints may present with major functional deficiency—due to persistence or localization—that is seen as a high HAQ value, which is one of the components of MDA (see above) [17]. In our study, the MDA components with the poorest results at the follow-up visits in patients with an oligoarticular pattern at diagnosis (compared with the polyarticular pattern) were global pain, patient global assessment of disease, number of painful entheses, and cutaneous involvement; this differences between both patterns were statistically significant at at least 1 of the 2 follow-up visits.

The remaining variables included in the model were less important for its predictions, and the direction of their association was less clear than for the previously mentioned variables. It is known that patients’ and physicians’ opinions with respect to disease activity and impact tend to be highly contradictory [18, 24, 25]. In addition, as we have already mentioned, the tender joint count is a component of the MDA score [4]. Finally, the potential association with weekly alcohol consumption is unclear and has not been identified in any studies on MDA. Alcohol consumption is clearly associated with an individual’s social and cultural setting, and we cannot generalize to settings other than those addressed in the present study. For example, in the CASCO cohort of Korean patients with axial spondyloarthritis, high socioeconomic status and alcohol consumption were associated with a lower Assessment of Spondyloarthritis International Society (ASAS) health index (lower disease impact) [26].

The main limitation of this study is its sample size and the fact that some data are missing for some variables. This affected the power of the statistical analysis and, therefore, the ability of the study to detect variables associated with the outcome measure. We tried to compensate for this by using models based on artificial intelligence and machine learning. Random forests are “joint” algorithms in which different decision trees are trained with different subsets of variables and data. Decision trees are more flexible than many statistical models, since they make it possible to identify many types of association between explanatory variables and the outcome measure. Furthermore, the fact that random forests add variability prevents the model from being overadjusted to the data and can be re-run with new data, thus increasing the robustness of the predictions.

The main strength of this study is its ability to record the course of PsA from an early phase before the natural disease evolution is modified by treatment prescribed by the rheumatologist.

## Conclusions

In this study of patients with recent-onset PsA, 4 of the 9 variables associated with the MDA response had a greater predictive ability based on our artificial intelligence model. These were global pain, impact of the disease (PsAID), patient global assessment of disease, and physical function (HAQ-Disability Index). Therefore, a key objective in the management of PsA should be control of pain, which is not always associated with inflammatory burden, and the establishment of measures—both generic and specific—to better control the various domains of PsA. While estimations on disease made by physicians and patients are often divergent, the patient’s opinion is key when sharing decision-making.

## Supplementary Information

**Additional file 1.** Explanation of the methods based on artificial intelligence used in the analysis.

## Data Availability

The datasets used and/or analyzed during the current study are available from the corresponding author on reasonable request to proyectos@ser.es.
