# Supplementary material for: Minimal disease activity (MDA) in patients with recent-onset psoriatic arthritis: predictive model based on machine learning
Source: Arthritis Res Ther. 2022 Jun 24;24:153. doi: 10.1186/s13075-022-02838-2 (PMC9229524; doi:10.1186/s13075-022-02838-2)

1. **Random forest:**

Random forests are extensions of decision trees. A decision tree model makes predictions by navigating nodes until a leaf node is reached. In order to construct a decision pathway, we must select the feature that best separates data items into 2 groups and construct an “X≤y” type condition. If a data item fulfills the condition when navigating through the tree, it takes the left branch; if it does not, it takes the right branch.

A random forest involves training a set of decision trees in which each tree votes on the classification of data. The final decision in this model is that voted for by most trees. For the decision trees in the set to be different, each tree is trained with different subsets of data and different groups of variables.

Once a random forest is trained, the importance that the model gives to each variable can be obtained based on the number of times the model uses a variable to establish a condition of the type "X <= y" normalized by the number of data that those conditions separate.

1. **XGBoost:**

XGBoost is another decision tree–based algorithm. Unlike the previous approach, which used randomization to improve individual models, models based on boosting use the predictions of each individual tree to improve the results of the following individual tree. Instead of training all the trees in parallel and predicting based on a vote, XGBoost links the results of a tree for sequential training of the following tree.

XGBoost differs from other decision tree boosting algorithms in that, for training, it uses the gradient descent technique to minimize error, regularization (limiting the number of trees and their depth) to ensure that the fit between the model and the training subset is not too close, and other optimization parameters to improve performance.

Gradient descent is an optimization algorithm. Optimization algorithms are used to minimize functions (ie, to find parameters in the model that minimize error between predictions and real data). Gradient descent progressively evaluates the function in an iterative fashion; it takes steps in the opposite direction to that of the gradient of the function at each point, since this is the steepest descent, until a minimum is found.

1. **SHAP:**

SHAP method is a technique for explaining the output of artificial intelligence models. It assigns a SHAP value to each value of each variable of each data item in the evaluation set according to the degree to which it affects the prediction of the model (the higher the SHAP value, the more the data item affects the prediction) and to the direction in which it affects the prediction of the model (a positive SHAP value indicates a positive effect on the prediction, that is, it contributes to the prediction having a higher value, and the opposite with a negative SHAP value).

Specifically, the method involves running the data item through the model, removing it from its trees, and calculating the SHAP value based on the differences in the predictions with and without the data item. It is important to understand that SHAP explains the model and not the values of the variables directly. That is, for the same value of a variable in different subjects or at different visits, the SHAP values may differ depending on the values of the other variables in each subject or at each visit.

The figure shows an example of SHAP plot. The Y axis represents the 20 most influential variables in the predictions of the model; the X axis shows the SHAP values (contribution) relative to the variables.

The point clouds represent all the data items in the set distributed according to their SHAP values; the colors symbolize the values of the variables, with red for high values and blue for low values.

The SHAP plot orders variables by their importance in the predictions of the model. This importance is calculated with the mean of the magnitudes of the SHAP values assigned to the variable.


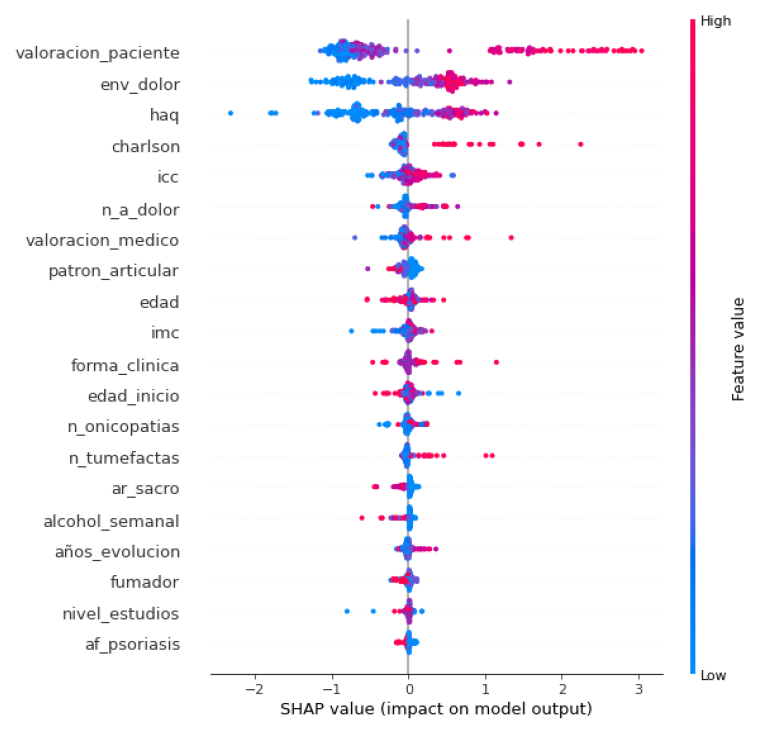

Supplement: Supplementary file 1 — Additional file 1. Explanation of the methods based on artificial intelligence used in the analysis. [file 13075_2022_2838_MOESM1_ESM.docx]
